# Mesenchymal Stromal Cell Healing Outcomes in Clinical and Pre-Clinical Models to Treat Pressure Ulcers: A Systematic Review

**DOI:** 10.3390/jcm12247545

**Published:** 2023-12-07

**Authors:** Ricardo A. Torres-Guzman, Francisco R. Avila, Karla Maita, John P. Garcia, Gioacchino D. De Sario, Sahar Borna, Abdullah S. Eldaly, Alfredo Quinones-Hinojosa, Abba C. Zubair, Olivia A. Ho, Antonio J. Forte

**Affiliations:** 1 Division of Plastic Surgery, Mayo Clinic, Jacksonville, FL 32224, USA; 2 Department of Neurosurgery, Mayo Clinic, Jacksonville, FL 32224, USA; 3 Department of Laboratory Medicine and Pathology, Transfusion Medicines and Stem Cell Therapy, Mayo Clinic, Jacksonville, FL 32224, USA

**Keywords:** animal model, pressure sores, tissue engineering, wounds and injuries

## Abstract

Background: Despite numerous measures used to prevent pressure ulcers, their growing prevalence in recent years is expected to continue as the population ages. This review aims to report the outcomes of the regenerative potential of MSCs in treating pressure ulcers, assessing the effectiveness of MSCs in treating pressure ulcers. Methods: A computerized search for articles on animal models that use MSCs as primary therapy to treat pressure ulcers, published from conception to present, was conducted using PubMed, MEDLINE, Embase, and CINAHL. Our search yielded 52 articles, narrowed to 44 after excluding duplicates. Results: Out of 52 articles collected from four databases, 11 met the inclusion criteria. A total of 11 articles published between 2008 and 2020 met the inclusion criteria. Eight studies were observational descriptive papers in animal models, and three were prospective. Six studies used autologous MSCs, while five used allogenic MSCs. Three studies were conducted in humans, and the remaining eight were conducted in animals. The most common method of cell delivery was an intradermal injection in the margins of the ulcer. All studies reported positive results, including improved wound healing, reduced inflammation, and improved tissue regeneration. Conclusions: MSCs have shown promising results in treating pressure ulcers in animal and clinical trials. The combination of MSCs and scaffold materials has also been studied and found to be effective in wound healing. A standardized human wound model has been proposed further to investigate the efficacy of cell-based therapies for chronic wounds. However, more research is needed to determine the best quantity of cells to apply for pressure ulcers and to ensure the safety and efficacy of these treatments in clinical settings.

## 1. Introduction

The cost of treating pressure ulcers in the US is USD 11 billion annually [1]. Furthermore, most lawsuits are brought about by inadequate prevention rules in favor of patients who require long-term care [2]. About 70% of pressure ulcers occur in patients who are 70 years old [3], with more cases likely as life expectancy increases. The overall estimate of pressure ulcers across all healthcare institutions in Canada is 26% [3]. The Agency for Healthcare Research and Quality reports that nearly 2.5 million people are diagnosed with pressure ulcers, and with more than 60,000 deaths caused by pressure ulcers yearly in the US [4]. The current standard of care for less severe ulcers (stages I and II) is conservative management; surgery and negative pressure are usually indicated for severe ulcers (stages III and IV) [1]. However, either some cases are resistant to treatment, or the healing process is slower than expected [5]. Given the background on current management and prevention strategies for pressure ulcers, new technologies and alternative treatments must be considered.

**Figure 1:**
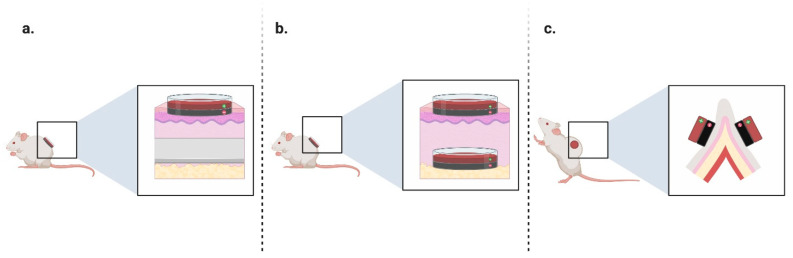
Timeline of pressure ulcer models. (**a**). Exhibits using a heavy metal plate intradermally and a magnet on the outside to reproduce a pressure ulcer. (**b**). Posteriorly, the heavy metal plate is replaced by another magnet. (**c**). Magnets are placed externally to avoid invasive procedures, having the same results as previous models. Created with BioRender.com.

Having a reliable animal model for pressure ulcers is crucial for effective pre-clinical research, enabling the evaluation of drug efficacy before human trials. It aids in comprehending ulcer formation and treatment impacts (see Figure 1). Prior models caused discomfort due to prolonged immobilization and anesthesia, pushing for advancements [6].

Several reports studying the tissue regenerative capacity of MSCs have shown promising outcomes, with clinical reports verifying the potential efficacy of this cell-based therapy alone or in combination with grafts to treat severe skin burns, radiation-related skin lesions, non-healing diabetic ulcers, and dystrophic epidermolysis bullosa, among others [7,8,9,10,11]. Highlighting the potential of xenogenic MSCs, particularly ADSCs and BMSCs, in treating chronic wounds such as pressure ulcers, radiation injuries, and burns, these studies suggest their valuable role in wound healing. Despite the low immunogenicity of MSCs, the risk of immunogenic reactions has constrained the literature reports on xenogenic MSC use [12]. ADSCs are preferred for pressure ulcer treatment due to their low expression of MHC class II and other costimulatory molecules, along with ethical feasibility, easy extraction, and a higher yield of cultured MSCs compared with BMSCs [13,14,15,16]. This review aims to report the outcomes of the regenerative potential of MSCs in treating pressure ulcers, assessing the effectiveness of MSCs in treating pressure ulcers.

## 2. Methods

### 2.1. Eligibility Criteria

Human and animal studies that used MSCs as primary therapy to prevent or treat pressure ulcers were included. Studies in languages other than English, reviews, conference articles, and book chapters were excluded.

### 2.2. Information Sources and Search Strategy

A computerized search was conducted on 10 April 2023 by the first and second authors independently using PubMed (1994–present), MEDLINE (1996–present), Embase (1988–present), Web of Science (1997–present), and CINAHL (1994–present). The following search strategy was used: (“Mesenchymal Stem Cells” OR “Bone Marrow Mesenchymal Stem Cells” OR “Adipose-Derived Mesenchymal Stem Cells” OR “Mesenchymal Stromal Cells” OR “Multipotent Mesenchymal Stromal Cells” OR “Mesenchymal Progenitor Cell”) AND (“Pressure Ulcers” OR “Bedsore” OR “Bedsores” OR “Pressure Sore” OR “Decubitus Ulcer”).

### 2.3. Study Selection and Data Collection Process

**Figure 2:**
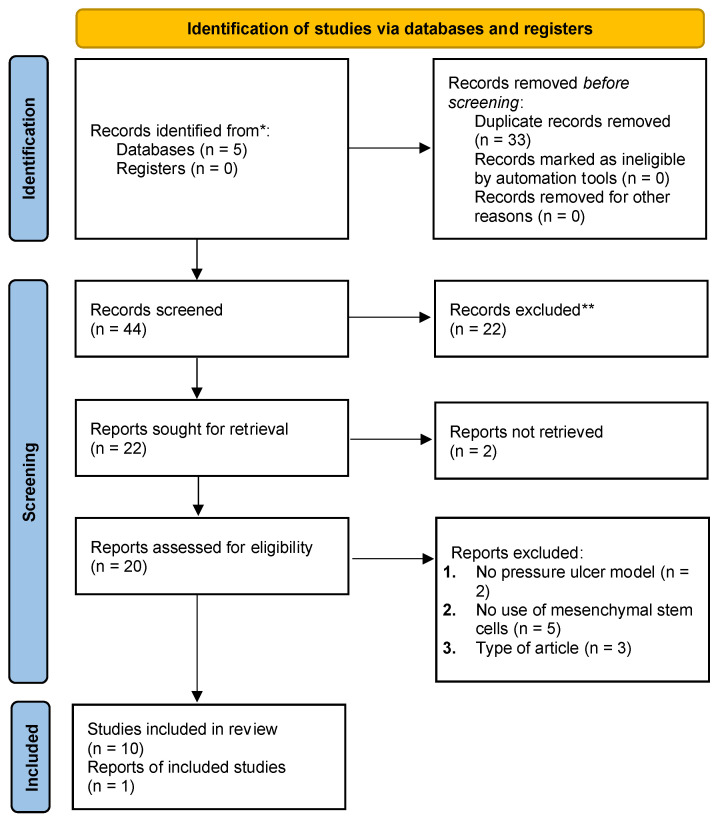
PRISMA 2020 flow diagram for new systematic reviews, which included searches of databases and registers only. * Consider, if feasible to do so, reporting the number of records identified from each database or register searched (rather than the total number across all databases/registers). ** If automation tools were used, indicate how many records were excluded by a human and how many were excluded by automation tools.

The first and second authors independently scanned and filtered through records based on the titles and abstracts in accordance with the inclusion and exclusion criteria mentioned earlier. Subsequently, the full-text reports were screened and filtered. Whenever the authors disagreed on whether to include or exclude a study, the senior author (A.J.F.) would provide the final decision. The filtration process of the studies is outlined in Figure 2.

### 2.4. Risk of Bias of Individual and Across Studies

**Figure 3:**
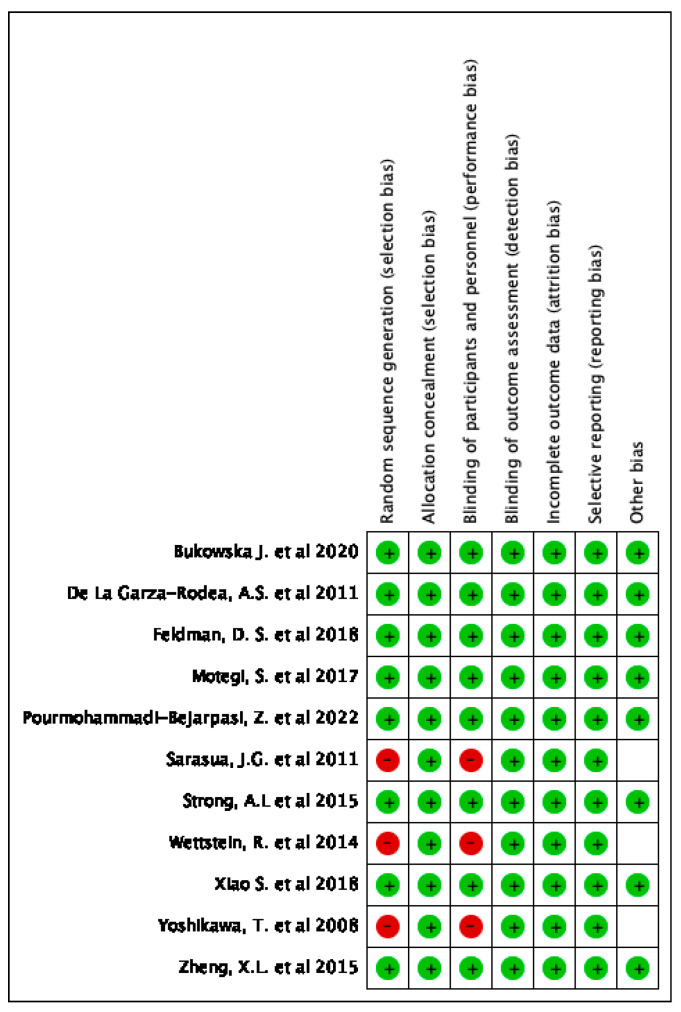
Individualized bias [5,13,17,18,19,20,21,22,23,24,25].

**Figure 4:**
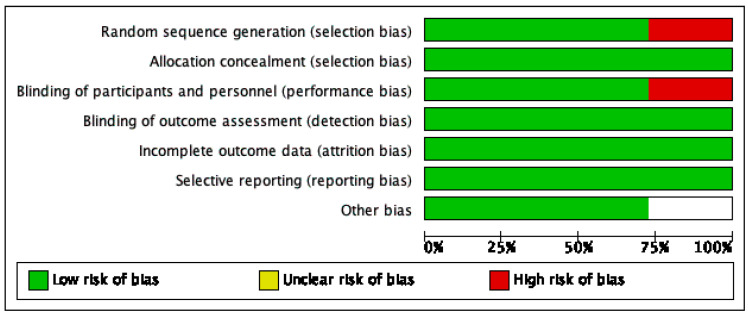
Cross studies bias [5,13,17,18,19,20,21,22,23,24,25].

The risk of bias of the studies selected was assessed with the help of ROBINS-I tool of the Cochrane Library for non-randomized studies. A description of individualized bias and cross-bias is shown in Figure 3 and Figure 4, respectively.

## 3. Results

### 3.1. Study Selection and Characteristics

Of the 52 articles from the four databases mentioned above, 11 met our inclusion criteria (see Table 1). Six studies used autologous MSCs, while five used allogenic MSCs. Six used bone-marrow-derived stromal cells (BMSCs), three used adipose-derived stromal cells (ADSCs), one used human umbilical cord MSCs (hUC-MSCs), and one used human amniotic epithelial cells (hAECs). Three studies were conducted in humans, and the remaining eight were conducted in animals, with five conducted in mice, two in rats, and one in rabbits. The most common method of cell delivery was an intradermal injection in the margins of the wound. Pre-clinical studies used between 1.0 × 10^5^ and 1.0 × 10^6^ cells. On the other hand, clinical studies used between 1.0 × 10^5^ and 60 million cells.

### 3.2. Use of MSCs to Treat Pressure Ulcers in Pre-Clinical Studies

The research investigating the use of *MSCs* to treat pressure ulcers is relatively new. In 2011, de la Garza-Rodea et al. [17] described the capacity of human BMSCs to aid the restorative process in a novel pressure ulcer model in mice. They used lentivirus to transfect the human BMSCs with b-galactosidase to track the cells and applied it posteriorly to the pressure ulcers, which had been irradiated to delay healing. Results showed no change in healing time or ulcer size compared with controls. Histologic examination of the epidermal layer, where healing was delayed with irradiation, exhibited no improvement with administering human BMSCs.

ADSCs were not studied in animal models as an alternative to BMSCs until 2015. Strong, A.L. et al. [13] tested the hypothesis that ADSCs can improve pressure ulcer repair in both young (2-month-old) and elderly (20-month-old) mice. The mice were given a pressure ulcer and injected with ADSCs the next day. ADSC-treated mice demonstrated cell-concentration-dependent wound closure, improved epidermal/dermal architecture, increased adipogenesis, and decreased inflammatory cell infiltration. Wounds treated with ADSCs were smaller in diameter size after 10 days (1.0 × 10^5^ ADSCs = 42.7 mm^2^; 2.5 × 10^5^ ADSCs = 43.3 mm^2^; 1.0 × 10^6^ ADSCs = 22.3 mm^2^) than wounds treated with PBS (55.9 mm^2^; *p*-value < 0.05). Higher concentrations of ADSCs (1.0 × 10^6^) supplied to the wound site hastened wound closure even more than lower concentrations, implying that higher dosages of ADSCs may be more effective at healing pressure ulcer wounds.

In the same year, Zheng X.L. et al. [18] aimed to see how hAEC transplantation affected the pathologic changes in wound tissues in a rat model of stage III pressure ulcer. Ninety-six rats were randomly assigned to one of four groups: model, hAEC transplantation, traditional treatment, and control. The healing time of the hAEC transplantation group (group H) was significantly shorter than the other three groups, and pathological observations revealed that group H exhibited significant proliferation of fibrous tissues and vessels in the dermal layer, as well as a significantly longer appearance time and degree of skin appendages than the other three groups. This study found that hAEC transplantation can significantly accelerate the healing of stage III pressure ulcers.

In 2017, Motegi, S. et al. [19] investigated the effect of MSC injection on the formation of cutaneous pressure ulcers following cutaneous ischemia-reperfusion (I/R) injury in 2017. Subcutaneous injection of MSCs into I/R injured skin significantly suppressed pressure ulcer formation by reducing vascular damage, oxidative cellular damage, oxidative stress, endoplasmic reticulum stress, and apoptosis. An MSC-conditioned medium also inhibited the generation of reactive oxygen species and apoptosis in cultured fibroblasts. These findings imply that MSC injection may protect against the development of pressure ulcers following cutaneous I/R injury.

Scaffold materials have been studied as a vehicle or even to improve the performance of MSCs. Feldman D.S. et al. [20] sought to assess the efficacy of a combination treatment for full-thickness wounds that included a biodegradable albumin scaffold, MSCs, and transforming growth factor B3 (TGF-3) overexpression. The research was carried out in an animal model to assess the feasibility of using the treatments alone or combined in a degradable regenerative system to meet the clinical performance design constraints. The objective was to determine how close the current system was to achieving the desired clinical performance. Researchers tested the efficacy of three healing strategies on full-thickness wounds: an albumin scaffold, MSCs, and TGF-3 overexpression. The findings indicated that at one-week post-surgery, the combined approach notably improved the healing rate compared with the control. Despite this, at two weeks post-surgery, the difference in healing rates was not statistically significant. The epithelialization rate (ER) was significantly better with the MSC-TGFβ3-A treatment, suggesting its effectiveness in tissue regeneration. However, the study did not find statistically significant differences in the contraction rate (CR) at the one-week or two-week time points.

Xiao et al. [5] explored the wound-healing properties of allogeneic MSCs in an animal model, comparing the healing outcomes of adipose-derived stem cells from diabetic (dhADSCs) and nondiabetic human adipose-derived stem cells (nhADSCs). They observed that the delivery of ASCs to the wound site accelerated wound healing in mice. Both nhADSCs and dhADSCs demonstrated improved healing over control treatments by day 5, with complete wound closure achieved by day 17. Importantly, in the first two weeks, the enhanced improvement in wound healing can be attributed to the effects of nhADSCs, which were more effective than dhADSCs in key healing processes such as reducing inflammation, promoting new blood vessel formation, and increasing collagen deposition. These processes are crucial during the early stages of wound healing, and nhADSCs particularly excel in these areas, leading to better outcomes in the initial healing phase. On days 9 and 13, mice treated with nhADSCs exhibited superior wound healing compared with those treated with dhADSCs, which was statistically significant (*p*-value < 0.005).

In 2020, Bukowska et al. [21] compared the effects of fresh and cryopreserved ADSCs in young and old mice, which were given intradermal injections and followed up daily for 20 days. Although neither fresh nor cryopreserved human ADSCs affected the healing process in older mice, younger mice exhibited accelerated and enhanced wound healing compared with controls. 

Pourmohammadi-Bejarpasi, Z. et al. [22] investigated the use of lipocalin 2 (Lcn2)-engineered MSCs and Metadichol (an inverse agonist of vitamin D receptor (VDR)) nano gel for the treatment of skin disorders such as burns, trauma, excisional and diabetic wounds, and bedsores in 2022. The effectiveness of the combination therapy was evaluated using an excisional wound rat model. The wound healing rate in the treatment group was significantly higher than that in the control group, with a wound repair rate of 95% 14 days after surgery and 100% after 21 days. According to the findings, combining Lcn2-engineered MSCs with Metadichol can be a promising therapeutic strategy for wound healing.

### 3.3. Use of MSCs to Treat Pressure Ulcers in Clinical Trials

Yoshikawa, T. et al. [23] investigated a new treatment technique for skin wounds that used BMSCs and an artificial dermis made of collagen sponge in 2008. A successful trial was conducted on 20 patients, with 18 achieving complete wound healing. The distribution of ulcers was as follows: decubitus ulcer (n = 11) (hip, n = 10; heel, n = 1) and skin ulcer of the lower leg or foot (n = 7) (trauma, n = 4; burn, n = 1; phlebitis of the lower leg, n = 2). The remaining two patients died from diseases unrelated to the grafting.

In 2011, Sarasua J. G. et al. [24] investigated using BMSCs to promote the healing of pressure ulcers in patients with spinal cord injury. The ulcers were debrided and treated with BMSCs in 22 patients with SCI and single type IV pressure ulcers that had been present for more than 4 months. After a mean of 21 days, 19 of the patients (86.36%) had fully healed pressure ulcers. The mean intra-hospital stay was reduced from 85.16 to 43.06 days compared with conventional surgical treatment. Following treatment, daily wound care took only 5 min per patient instead of 20 min with conventional surgery. During a 19-month average follow-up, none of the resolved ulcers recurred. These findings suggest that cell therapy with autologous BMSCs can be an effective treatment option for type IV pressure ulcers in spinal cord injury patients, avoiding major surgical interventions.

Wettstein R. et al. [25] presented a standardized human wound model in 2014 to investigate the first three weeks following cell-based therapy for chronic wounds. The model was validated by injecting a CD34+ selected cell suspension into three patients’ wounds and measuring wound volume and histology before reconstructing the defect with a local flap. The wound volume was reduced to 60% of baseline on the sham side and 52% on the cell side, with no signs of metaplastic, dysplastic, or neoplastic proliferation/differentiation. CD34+ cells were also found in day 0 biopsies. The model is useful because it allows for objective outcome analysis with minimal additional morbidity, and the anatomy of the sacral area allows for a control and study side to be performed on the same patient.

## 4. Discussion

### 4.1. MSC Mechanisms in the Treatment of Pressure Ulcers

Pressure ulcers are a type of injury that occurs when an area of skin is subjected to sustained pressure for an extended length of time, resulting in tissue ischemia, a halt of nourishment and oxygen delivery to the tissues, and eventually tissue necrosis. Pressure ulcers can be defined as localized, acute ischemic damage to any tissue induced by applying external force [27].

Pressure ulcers, though extensively addressed for prevention, persist as a prevalent issue in healthcare and community settings, often leading to chronic and recurrent conditions. Thus, informed healthcare personnel must prioritize prevention strategies, including repositioning, to mitigate prolonged pressure effects. Risk assessment should consider factors such as inclined angle and positioning [28]. Given the limitations of current interventions, exploring alternative treatments, such as mesenchymal stem cells, is crucial, particularly in the context of this manuscript’s study focus.

A critical decision point in MSC therapy revolves around the choice between autologous and allogeneic sources. While autologous MSCs offer immune compatibility, their availability can be constrained by patient-specific factors. In contrast, allogeneic MSCs provide a readily accessible option but raise concerns about potential immune reactions and graft-versus-host responses. A study by Schatteman and Ma [29] reports outcomes that suggest an advantage of BMSCs from younger donors over those of older donors to promote wound healing of pressure ulcers by promoting angiogenesis and increasing wound closure rates. Similarly, ADSCs secrete paracrine growth factors, such as hepatocyte growth factor (HGF) and vascular endothelial growth factor (VEGF), which contribute to vascularization and angiogenetics [30], and cytokines able to induce tissue regeneration [31,32]. Moreover, hypoxemic preconditioning of MSCs before use has shown angiogenesis and accelerated damage repair in pressure ulcers [33] and infarcted myocardium [34].

Studying pressure ulcers in animal models is challenging due to the vast heterogenicity of patient populations in terms of etiology, comorbidities, size, localization, and even social environment. de la Garza-Rodea et al. [17] hypothesized that pressure ulcers have a delayed wound healing process and designed a model to observe the process of that behavior in mice.

To simulate this condition, they analyzed the effects of healing in diabetic mice and mice that had undergone irradiation to delay healing. No delay in healing was reported in the diabetic mice compared with the nondiabetic mice, in contrast to what has been reported in other studies that used surgical excisional skin wounds instead of pressure ulcer models [35,36,37,38].

It is known that diabetes is capable of blunting the regenerative characteristics of ADSCs [39,40] by flaws in oxidative stress and autophagy [41,42]. Moreover, diabetes and metabolic syndrome can alter the vital cell count, senescence, and oxidative stress in ADSCs [43].

On the other hand, the study results conducted by de la Garza-Rodea et al. [17] demonstrated that skin irradiation had prolonged epidermal repair and prevented complete regeneration of the dermis and hypodermis in a minimum period of 3 months.

Understanding the comparative efficacy and safety of these approaches is essential for optimizing treatment strategies and enhancing the clinical application of MSC-based interventions for pressure ulcer management. The results presented in this study provide comprehensive insights into the healing potential of MSCs in the context of pressure ulcers in animal models. Through a series of experiments conducted by various research groups, multiple aspects of the healing process, including angiogenesis, neurogenesis, osteogenesis, and adipogenesis, were evaluated [5,13,19,21].

Xiao S. et al. [5] demonstrated that both freshly isolated and cryopreserved adipose-derived stem cells (cADSCs) exhibited significant wound healing capabilities, with the trend persisting until complete wound closure was achieved on day 17. Notably, the superiority of freshly isolated ASCs over cryopreserved ones was observed at specific time points, suggesting the potential influence of storage methods on the therapeutic efficacy of ASCs. The study’s findings indicate that on day 21, the use of cADSCs led to significantly increased epidermal thickness and collagen accumulation, with the most pronounced effects observed in the ndADSC group. Notably, dADSCs exhibited some impairment in processes crucial to wound healing, including angiogenesis and re-epithelialization. However, they still demonstrated beneficial impacts, particularly in promoting early-stage wound healing and stimulating angiogenesis and collagen production. Previous research [44,45] suggests that preconditioning dADSCs with antioxidants can reverse their compromised properties, emphasizing a promising avenue for enhancing their therapeutic efficacy in clinical applications.

It is critical to emphasize the disparities in efficacy seen among studies. The study by Xiao et al. [5], for example, indicated a significantly quicker healing rate employing ADSCs from both diabetes and nondiabetic persons. nhADSCs showed improved regenerative ability, significantly impacting critical healing processes such as inflammation reduction, angiogenesis, and collagen deposition, resulting in wound closure by day 17. Pourmohammadi et al.’s trial [22], which used a combination therapy of Lcn2-engineered MSCs and Metadichol, achieved a 95% repair rate by day 14 and complete healing by day 21. The disparity in healing rates can be attributable to different mechanisms of action and the nature of the therapies used. While ADSCs directly impact crucial early-stage healing processes, the combination therapy in the Pourmohammadi et al. [22] study might have had a more gradual yet effective healing impact. These observations underscore the potential of MSCs in wound healing, especially for pressure ulcers, and highlight the importance of considering the type of MSCs and the therapeutic approach in designing treatment strategies. However, it is essential to cautiously approach direct comparisons due to potential variations in study designs, methodologies, and experimental models.

Similarly, Bukowska et al. [21] examined the PU healing response in young and older mice, revealing age-related variations in the response to ASC therapy. While younger mice displayed accelerated wound healing with ASC treatment, older mice did not experience significantly faster closure than controls. The findings emphasize the complexity of MSC-based interventions, particularly in older subjects, highlighting the need for tailored treatment strategies for different age groups.

Contrasting these findings, the study by De la Garza et al. [17] emphasized the critical role of ischemia-reperfusion injury in PU formation, indicating a clear correlation between the duration of ischemia and the subsequent healing rates. The study highlighted the differences in wound progression in hairy and hairless mice, suggesting a nuanced relationship between ischemic duration and wound-healing dynamics.

MSCs promote angiogenesis by developing into endothelial cells and releasing growth factors and chemokines that boost host endothelial cell recruitment and proliferation [46]. Motegi’s study [19] revealed that MSCs, especially when preconditioned under hypoxic conditions, significantly protected against PU formation in a mouse model of cutaneous ischemia-reperfusion injury. The findings underscore the importance of appropriate preconditioning methods in enhancing the therapeutic potential of MSCs, emphasizing their role in mitigating vascular damage associated with cutaneous ischemia-reperfusion injury.

Moreover, the investigation conducted by Strong et al. [13] highlighted the dose-dependent effects of ASCs on wound healing, with higher concentrations of ADSCs demonstrating enhanced wound closure in young mice. The study also emphasized the efficacy of ADSCs in promoting wound healing in both young and older mice, indicating their potential for use across diverse age groups.

Furthermore, the evaluation of the angiogenesis, neurogenesis, osteogenesis, and adipogenesis outcomes of MSCs in PU healing, as elucidated by Xiao S. et al., Budkowska, Motegi, and Strong [5,13,19,21], provided insights into the diverse mechanisms underlying the therapeutic effects of MSCs. These studies collectively suggest the potential of MSCs in modulating various cellular pathways to promote efficient wound healing and tissue regeneration.

When discussing the application of MSCs in treating pressure ulcers, it is essential to consider both their biodistribution and the uncertainties surrounding their potential for immunological rejection. The biodistribution of MSCs significantly varies with the mode of administration. Intravenous injection, the most prevalent method, typically results in MSCs being retained mostly in the lungs before a limited redistribution to organs like the liver, spleen, and kidneys. In contrast, intraarterial injection bypasses the pulmonary filter, allowing for greater dispersion, particularly in the irrigated area of the cannulated artery. Localized injections, such as intramuscular, intraarticular, and intralesional, lead to MSCs remaining primarily at the injection site, which can be advantageous for targeted therapy in pressure ulcers. Additionally, when MSCs are injected into specific organs or anatomical cavities, they disseminate in alignment with the natural flow of those systems [47].

Simultaneously, there is a need to address the potential immunological rejection of MSCs. Despite their recognized immunomodulatory properties, recent studies indicate that mismatched MSCs can be immunogenic, leading to complications like graft failure and transplant rejection. Data show that a significant proportion of patients treated with allo-MSCs develop donor-specific antibodies, with about 11.51% exhibiting this response post-treatment. However, this immunogenic reaction has not been definitively linked to decreased safety or tolerability of the treatment. These insights underscore the necessity for further research to fully understand the therapeutic implications of MSCs in treating pressure ulcers, particularly considering the potential for immune rejection and its impact on treatment efficacy. This dual focus on MSC biodistribution and immunogenicity will be critical in optimizing MSC-based therapies for effective pressure ulcer treatment [48].

### 4.2. Limitations

There need to be more data in the literature regarding the study of pressure ulcers, of which only studies published in English were included in our review. Additionally, there needs to be a standardized technique to study pressure ulcers, and the potential bias of misinterpreting data and results creates new variables to consider.

## 5. Conclusions

The comprehensive analysis of 11 articles revealed the significant potential of MSC therapies, particularly ADSCs and BMSCs, in treating pressure ulcers. These therapies have shown promising results in both pre-clinical and clinical settings, facilitating wound closure and tissue regeneration. Notably, the intricate interplay of MSC mechanisms in promoting angiogenesis, neurogenesis, osteogenesis, and adipogenesis underscores their versatility in addressing various cellular pathways for effective wound healing. However, the need for standardized research techniques and the potential for data misinterpretation necessitate further exploration and standardization in this field.

## Figures and Tables

**Table 1:** Summary of included studies.

Model/Participants	Animal	Type of Study	Type of Cell	Number of Cells Administered	Method of Cell Administration	Reference
19 men and 3 women with type IV chronic pressure ulcers; mean age: 56.41 years (range 29–79 years)	Human	Clinical	Human BMSCs	≈60 million cells	Intradermal injection in ulcer margins	[24]
9 men and 11 women; average age, 64.8 years; range, 22 to 91 years (n = 20)	Human	Clinical	Human BMSCs	1.0 × 10^5^ cells/mL	Artificial dermis composite graft	[23]
Complete para- or tetraplegic patients with a primary sacral pressure sore grade III–IV (n = 36)	Human	Clinical	hBMSCs	Between 17.4 × 10^6^ cells and 25.5 × 10^6^ cells/mL	Intradermal injection half of the ulcer	[25]
Female C57BL/6 wild-type mice (aged 6–8 weeks) n = 24	Mice	Pre-clinical	Human ADSCs	1.0 × 10^6^ cells	Intradermal injection in ulcer margins	[5]
Male and female C57BL/6 mice (aged 8 weeks to 22 months old)	Mice	Pre-clinical	Human ADSCs	Not stated	Intradermal injection in ulcer margins	[21]
Male and female NOD/SCID mice (aged 8–12 weeks).	Mice	Pre-clinical	Human BMSCs	5.0 × 10^5^ cells	Intradermal injection in ulcer margins	[17]
OKD48 mice with a transgene encoding a modified nuclear factor (erythroid-derived 2)-related factor 2 (Nrf2) protein	Mice	Pre-clinical	BMSCs	2.0 × 10^6^ cells/200 μL	Intradermal injection in ulcer margins	[19]
Female C57BL/6 wild-type mice	Mice	Pre-clinical	ADSCs	Between 1.0 × 10^5^ cells and 1.0 × 10^6^ cells	Intradermal injection in ulcer margins	[13]
New Zealand White rabbits weighing approximately 3 kg.	Rabbit	Pre-clinical	BMSCs	4.0 × 10^5^ cells/mL	Intradermal injection in ulcer margins	[26]
Male Wistar rats weighing 200–220 g (n = 74)	Rats	Pre-clinical	hUC-MSCs	1.0 × 10^6^ cells	Intradermal injection in ulcer margins	[22]
Male Sprague–Dawley rats, weighing between 120 and 150 g with stage III pressure ulcers (n = 96)	Rats	Pre-clinical	hAECs	5.0 × 10^5^ cells/mL	Subcutaneous injection	[18]

Abbreviations: ADSCs, adipose-derived stromal cells; BMSCs, bone marrow stromal cells; hAECs, human amniotic epithelial cells; hUC-MSCs, human umbilical cord mesenchymal stromal cells.

## Data Availability

Not applicable.

## Abbreviations

*MSC*	Mesenchymal stromal cell
*BMSC*	Bone-marrow-derived stromal cell
*ADSC*	Adipose-derived stromal cell
*cADSCs*	Cryopreserved adipose-derived stem cells
*hUC-MSCs*	Human umbilical cord mesenchymal stromal cells
*hAEC*	Human amniotic epithelial cell
*I/R*	Ischemia-reperfusion
*TGF-3*	Transforming growth factor B3
*dhADSC*	Human adipose-derived stromal cells extracted from diabetic subjects
*nhADSCs*	Human adipose-derived stromal cells extracted from nondiabetic subjects
*Lcn2*	Lipocalin 2
*VDR*	Vitamin D receptor
*HGF*	Hepatocyte growth factor
*VEGF*	Vascular endothelial growth factor
*TNF*	Tumor necrosis factor
*TSG-6*	Tumor necrosis factor stimulated gene-6
*IL-1RA*	Interleukin-1 receptor antagonist
*NFkB*	Nuclear factor kappa B
